# Maternal Obesity Affects Fetal Neurodevelopmental and Metabolic Gene Expression: A Pilot Study

**DOI:** 10.1371/journal.pone.0088661

**Published:** 2014-02-18

**Authors:** Andrea G. Edlow, Neeta L. Vora, Lisa Hui, Heather C. Wick, Janet M. Cowan, Diana W. Bianchi

**Affiliations:** 1 Mother Infant Research Institute, Tufts Medical Center, Boston, Massachusetts, United States of America; 2 Department of Computer Science, Tufts University, Medford, Massachusetts, United States of America; 3 Department of Pathology and Laboratory Medicine, Tufts Medical Center, Boston, Massachusetts, United States of America; Virgen Macarena University Hospital, School of Medicine, University of Seville, Spain

## Abstract

**Objective:**

One in three pregnant women in the United States is obese. Their offspring are at increased risk for neurodevelopmental and metabolic morbidity. Underlying molecular mechanisms are poorly understood. We performed a global gene expression analysis of mid-trimester amniotic fluid cell-free fetal RNA in obese versus lean pregnant women.

**Methods:**

This prospective pilot study included eight obese (BMI≥30) and eight lean (BMI<25) women undergoing clinically indicated mid-trimester genetic amniocentesis. Subjects were matched for gestational age and fetal sex. Fetuses with abnormal karyotype or structural anomalies were excluded. Cell-free fetal RNA was extracted from amniotic fluid and hybridized to whole genome expression arrays. Genes significantly differentially regulated in 8/8 obese-lean pairs were identified using paired t-tests with the Benjamini-Hochberg correction (false discovery rate of <0.05). Biological interpretation was performed with Ingenuity Pathway Analysis and the BioGPS gene expression atlas.

**Results:**

In fetuses of obese pregnant women, 205 genes were significantly differentially regulated. *Apolipoprotein D*, a gene highly expressed in the central nervous system and integral to lipid regulation, was the most up-regulated gene (9-fold). Apoptotic cell death was significantly down-regulated, particularly within nervous system pathways involving the cerebral cortex. Activation of the transcriptional regulators estrogen receptor, *FOS*, and *STAT3* was predicted in fetuses of obese women, suggesting a pro-estrogenic, pro-inflammatory milieu.

**Conclusion:**

Maternal obesity affects fetal neurodevelopmental and metabolic gene expression as early as the second trimester. These findings may have implications for postnatal neurodevelopmental and metabolic abnormalities described in the offspring of obese women.

## Introduction

Maternal obesity is a major public health problem in the United States. Sixty percent of reproductive age women are overweight at conception and one third are obese [1], [2]. There has been a parallel rise in childhood obesity and metabolic syndrome. This has coincided with an increased interest in the impact of the intrauterine environment on fetal gene expression and development [3]. Offspring of obese parents are significantly more likely to be obese and to have metabolic syndrome [4], [5]. Importantly, maternal body mass index (BMI) is more significantly associated with offspring obesity than paternal BMI, suggesting that the *in utero* environment plays an important role [6].

Maternal obesity also appears to have intergenerational health consequences beyond childhood metabolic syndrome and obesity. Data from large epidemiologic studies suggest an association with adverse neurodevelopmental outcomes in offspring, including lower general cognitive capabilities [7], [8], [9], and an increased incidence of autism spectrum disorders [10], developmental delay [11], and attention deficit hyperactivity disorder [12]. The molecular mechanisms by which maternal obesity might result in an increased risk for childhood obesity, metabolic syndrome, and adverse neurodevelopmental outcomes are currently unknown.

Amniotic fluid supernatant (AFS) offers unique advantages in studying real-time human fetal physiology and development. The analysis of cell-free fetal RNA (cffRNA) in AFS makes use of a readily available, typically discarded human biofluid. Prior work by our laboratory has demonstrated that fetal gene expression patterns in AFS vary according to gender, gestational age, and disease state [13], [14], [15], [16], [17]. Cell-free fetal nucleic acids are present in significantly higher concentrations in amniotic fluid, and arise from a distinct pool, compared to cell-free nucleic acids in maternal serum [18], [19], [20], [21]. While cell-free fetal DNA and RNA in maternal serum are known to arise from the placenta [22], [23], [24], [25], epigenetic studies and gene expression microarrays of cell-free fetal nucleic acids in amniotic fluid demonstrate relatively little contribution from the placenta [20], [25]. Thus, cell-free nucleic acids in amniotic fluid provide real-time information about fetal development.

Characterization of the normal second trimester amniotic fluid core transcriptome has demonstrated cffRNA transcripts in mid-trimester amniotic fluid reflecting the development of multiple organs including the fetal thyroid, liver, lung, pancreas, blood, and brain [14]. In prior work from our group, twenty-three highly organ-specific transcripts were identified, one-third of which mapped to the fetal brain [14]. This unexpected and novel finding has been substantiated in later studies [17], and suggests that amniotic fluid supernatant may be used to obtain neurodevelopmental information from living fetuses. These nucleic acid transcripts may pass into amniotic fluid via transport through fetal membranes in the fontanelle, nose, or ear; via shedding through urine; the trachea; fetal blood, or other mechanisms.

Here, we performed a discovery-driven research study to test the hypothesis that fetuses of obese women have different gene expression patterns than fetuses of lean women. We used cell-free RNA isolated from second trimester amniotic fluid supernatant, gene expression microarrays, and publicly available bioinformatics resources to identify differentially expressed genes, and their functions and interactions. In so doing, we have identified mechanisms that may be associated with an increased risk of neurodevelopmental and metabolic morbidity in offspring of obese pregnant women.

## Materials and Methods

### Ethics statement

Samples were collected with approval from the Tufts Medical Center Institutional Review Board from June 2011 through April 2012 (IRB protocol # 5582). Subjects signed informed consent for amniocentesis, which was performed for standard clinical indications.

### Recruitment and sample collection

**Figure 1:**
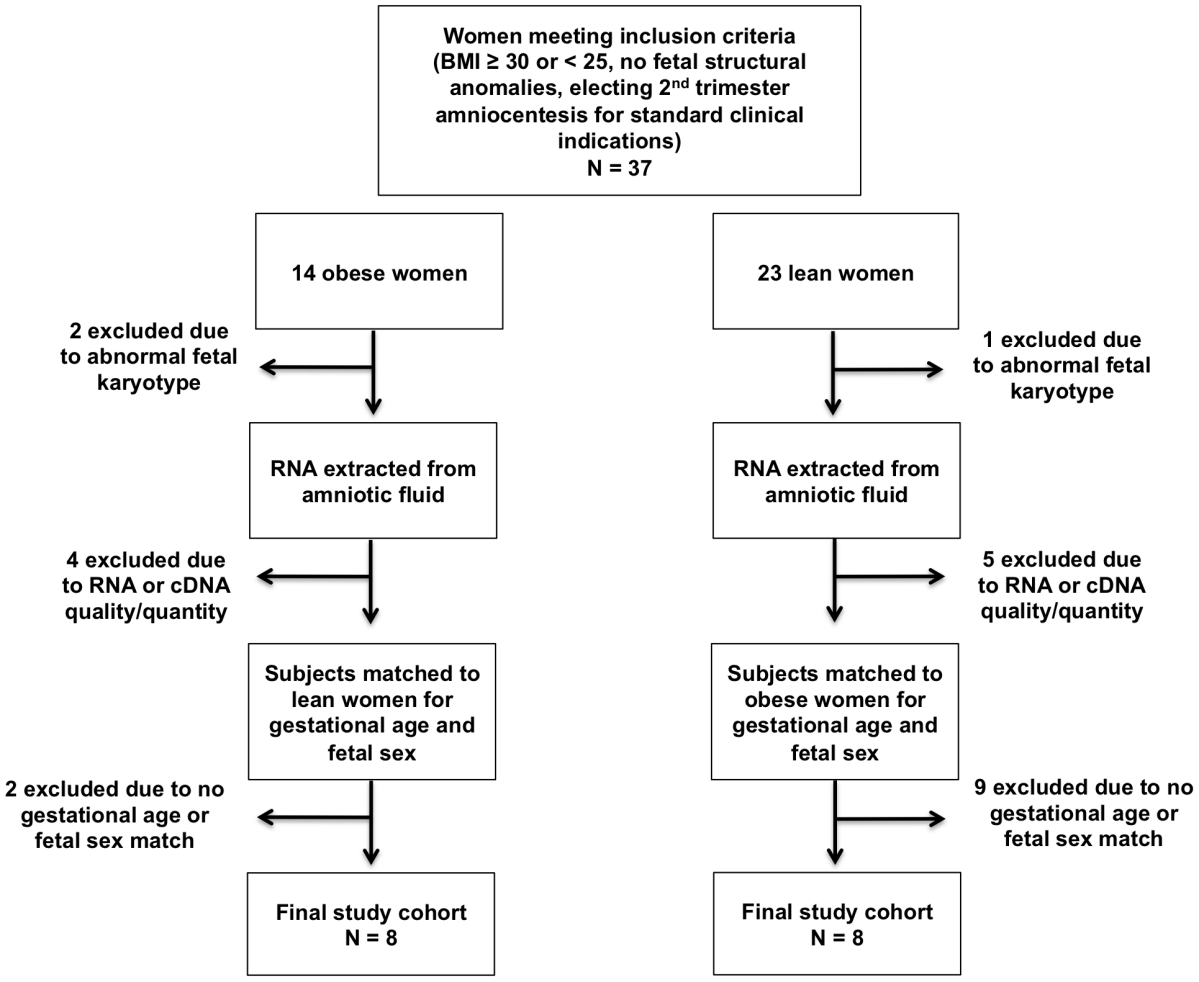
Flow of Subjects Through the Study. Process by which the final subjects were selected for microarray analysis.

This was a prospective pilot study of women with singleton fetuses without structural anomalies undergoing second trimester (14–24 weeks) genetic amniocentesis at Tufts Medical Center. Subjects signed informed consent for amniocentesis, which was performed for standard clinical indications (advanced maternal age, ultrasonographic soft markers of aneuploidy, abnormal serum screening results, or maternal request). Women with a BMI≥30 (obese, n = 14) or <25 (lean, n = 23) at the time of amniocentesis were enrolled. Per protocol, access to the medical record was limited to clinical information available at the time of amniocentesis (i.e. indications for the procedure, presence/absence of fetal anomalies, standard maternal demographic data), and cytogenetic results. Fetuses later found to have an abnormal karyotype, or those with RNA or cDNA of insufficient quality or quantity to hybridize to microarrays, were subsequently excluded. We aimed for a target of at least eight samples per group, based on the demonstration that near-maximal levels of statistical stability are obtained with between eight and 15 biological replicates in microarray studies [26]. Figure 1 depicts the flow of subjects through the study. Samples were matched for gestational age and fetal sex, both of which have been previously shown to influence fetal gene expression [13], [27]. The amniotic fluid samples were centrifuged at 165× g for 10 minutes at room temperature to separate amniocytes for subsequent diagnostic testing. The residual AFS was stored at −80°C until RNA extraction.

### RNA extraction, processing, and hybridization to microarrays

Cell-free fetal RNA was extracted from five to ten milliliters of AFS within six months of collection, according to previous protocols developed by our group to maximize RNA yield [28]. RNA was stored at −80°C for less than 6 months, to maximize RNA integrity [29]. Subjects were matched for gestational age, to avoid any confounding effects of gestational age on cell-free RNA quantity [13], [30]. RNA was extracted, purified, converted to cDNA, and hybridized to whole genome expression arrays (Affymetrix GeneChip® Human Genome U133 Plus 2.0, Affymetrix Inc.), using previously described protocols that have been established in our laboratory [31], [32].

### Gene expression data analysis

The gene expression array results have been uploaded to the Gene Expression Omnibus repository (GSE48521). The microarray data were normalized using the three-step command from the affyPLM package in Bioconductor [33], using ideal-mismatch background-signal adjustment, quantile normalization, and the Tukey biweight summary method [34]. This summary method includes a logarithmic transformation to improve the normality of the data. The mas5calls function from the Bioconductor affy package was used to obtain detection calls consistent with those produced by the Affymetrix 5.0 software. We used paired *t*-tests to identify differentially regulated probe sets in the fetuses of obese women compared to lean controls. The Benjamini-Hochberg (BH) correction was applied to the normalized expression data for all probe sets on the microarray, to adjust for multiple comparisons in order to limit the false discovery rate [35]. Throughout the manuscript, the terms “BH p-value” and “false discovery rate” are used interchangeably. A principal component analysis was performed using R (version 2.13.1) to identify dominant sources of variation in the gene expression data [36]. Boxplots were generated in the R software environment (version 2.13.1) to examine the distribution of normalized gene expression data across samples, and in obese versus lean study subjects.

### Functional genomic analysis

We considered genes that were up- or down-regulated in eight of eight pairs, and associated with BH-p values <0.05, to be significantly differentially regulated. The Affymetrix gene probe IDs, with corresponding median fold change and BH-p values, were uploaded to Ingenuity® Pathways Analysis (IPA, Ingenuity® Systems, Redwood City, CA version 9.0, content version 12710793). IPA utilizes a manually curated database containing biological interactions and functional annotations to identify differentially represented biological functions and/or diseases in a data set. IPA uses a right-tailed Fisher's exact test to calculate a significance score for each association between genes in the experimental dataset and a biological function. To reduce false positive results, we considered IPA pathways to be significant only if they were associated with a BH-p value <0.05. Within these pathways, we considered functional annotations associated with right-tailed Fisher's exact p-values <0.01 to reflect a statistically significant, non-random association. This cutoff is more stringent than the recommended threshold of <0.05 [37]. False-discovery rates, and when calculated, bias-corrected Z-scores (predicting the impact of gene expression on a particular function), were reported separately for each association. Only those functional annotations associated with three or more genes in the dataset were considered. Enriched pathways for the subcategories “Molecular and Cellular Functions,” and “Physiological System Development and Function” were reported separately.

The Upstream Regulator Analysis feature of IPA was utilized to predict the activation or inhibition of transcriptional regulators based on the direction of gene expression changes in our data set. We defined upstream regulators as significantly activated or inhibited if the bias-corrected Z-score was ≥2.0 or ≤2.0, respectively, in accordance with recommended thresholds [38].

We used a publicly-available gene expression atlas (http://biogps.gnf.org) to determine whether genes that were significantly dysregulated in fetuses of obese women had tissue-specific expression [39]. We chose the BioGPS atlas due to its coverage of normal adult and fetal tissues, compatibility with the Affymetrix microarray platform, and good correlation between transcript levels and protein abundance [40]. We considered genes to be highly organ-specific if they corresponded to a single organ with an expression value >30 multiples of the median (MoM), consistent with previously-established stringency thresholds [41].

## Results

The lean and obese study groups did not differ significantly with respect to maternal or gestational ages, but did vary significantly with respect to maternal BMI (Table 1). Clinical indications for amniocentesis were the same in both groups (advanced maternal age, abnormal serum screening result, and/or the presence of soft markers for aneuploidy on Level II ultrasound examination, Table 2). Mean array hybridization efficiency was similar, at 42.13%±3.54% in obese and 41.85%±4.92% in lean subjects.

**Table 1:** Demographic characteristics of obese and lean subjects.

Demographic characteristic	Obese	Lean	P-value
BMI, mean (SD) [range], kg/m^2^,	35.16 (3.20) [30.47–39.71]	21.96 (1.71) [18.76–24.05]	<0.001
Maternal age, mean (SD) [range], years	37.13 (5.0) [31–46]	33.88 (5.57) [23–39]	0.30
Gestational age, mean (SD) [range], weeks	17.86 (1.71) [15.86–20.14]	18 (1.40) [16.14–20.57]	0.73
Fetal Sex (number of males, number of females)	4, 4	4, 4	N/A

**Table 2:** Clinical characteristics of obese and lean subjects.

Obese Subjects				
Maternal age	GA weeks	Fetal sex	Maternal BMI (kg/m^2^)	Indications for amniocentesis
46	15 6/7	F	34	Abnormal serum screening result (increased risk trisomy 21)
38	16 2/7	M	33	Advanced maternal age
31	19 2/7	F	40	Sonographic soft marker (echogenic intracardiac focus)
37	16	M	30	Abnormal serum screening result (increased risk trisomy 21)
37	17 5/7	M	36	Sonographic soft markers (enlarged nuchal fold, shortened nasal bone)
42	20 1/7	F	33	Abnormal serum screening result (increased risk trisomy 18); sonographic soft marker (choroid plexus cyst)
33	19 5/7	F	35	Abnormal serum screening result (increased risk trisomy 21)
33	17 6/7	M	40	Abnormal serum screening result (increased risk trisomy 21); sonographic soft marker (echogenic intracardiac focus)

Lean Subjects				
Maternal age	GA weeks	Fetal sex	Maternal BMI (kg/m^2^)	Indications for amniocentesis
39	16 5/7	F	19	Advanced maternal age; sonographic soft marker (echogenic intracardiac focus)
34	16 1/7	M	21	Advanced maternal age; sonographic soft marker (echogenic intracardiac focus)
39	18	F	24	Advanced maternal age
23	17 1/7	M	24	Abnormal serum screening result (increased risk trisomy 21)
34	18 6/7	M	21	Sonographic soft marker (choroid plexus cyst)
36	20 4/7	F	23	Advanced maternal age
37	18 4/7	F	23	Advanced maternal age, sonographic soft marker (absent/hypoplastic nasal bone)
29	17 4/7	M	22	Abnormal serum screening result (increased risk trisomy 21)

The Affymetrix HG U133 Plus 2.0 array contains more than 54,000 probe sets, allowing for comprehensive analysis of whole genome expression. IPA analysis demonstrated differential regulation of 205 genes in fetuses of obese compared to lean women. One hundred and fourteen of these were significantly up-regulated, and 91 were significantly down-regulated (Table S1). These 205 genes comprise approximately one percent of the total number of unique genes interrogated by the Affymetrix whole genome array [42]. *Apolipoprotein D* (*APOD*) was the most up-regulated gene in fetuses of obese women (nine-fold). The top ten most up- and down-regulated genes in fetuses of obese women and their functions are listed in Tables 3 and 4.

**Table 3:** Top ten most up-regulated genes in fetuses of obese versus lean women in the second trimester.

Gene Name	Symbol	Gene Function*	Fold Change	BH p-value†
*Apolipoprotein D*	*APOD*	Encodes component of high density lipoprotein (HDL); lipocalin involved in lipid metabolism; response to reactive oxygen species; response to axon injury	9.2	0.03
*F-box and leucine-rich repeat protein 6*	*FBXL6*	Member of F-box protein family; protein ubiquitination; proteolysis	8.3	<0.001
*Synaptotagmin XIII*	*SYT13*	Member of the large synaptotagmin protein family; synaptotagmins function in vesicle-mediated transport	8.2	<0.001
*B-cell CLL/lymphoma 2*	*BCL2*	Outer mitochondrial membrane protein that blocks apoptotic cell death; overexpression causes follicular lymphoma; regulator of intrinsic apoptotic pathway; axon regeneration; brain development; immune system development	7.6	<0.001
*Matrix metallopeptidase 9 (gelatinase B, 92 kDa gelatinase, 92 kDa type IV collagenase)*	*MMP9*	Breakdown of extracellular matrix in normal and disease processes; degradation of Type IV and V collagens	5.8	<0.001
*Chromosome X open reading frame 56*	*CXorf56*	Function currently unknown	5.1	0.01
*MORN repeat containing 1*	*MORN1*	Function currently unknown	5.0	0.01
*Zinc finger protein 551*	*ZNF551*	Nuclear protein involved in DNA and metal ion binding; DNA-dependent transcription regulation	4.6	0.01
*Zinc finger protein 483*	*ZNF483*	Nuclear protein involved in DNA and metal ion binding; DNA-dependent transcription regulation; viral reproduction	4.3	<0.001
*B-cell CLL/lymphoma 3*	*BCL3*	Transcriptional co-activator; associates with NF-kappa B homodimers	4.3	<0.001

* Gene functions obtained from public databases (Entrez Gene and UniProt KB), descriptions modified due to space constraints.

† Synonymous with the false discovery rate.

**Table 4:** Top ten most down-regulated genes in fetuses of obese versus lean women in the second trimester.

Gene Name	Symbol	Gene Function*	Fold Change	BH p-value†
*Serine/threonine kinase 24*	*STK24*	Serine/threonine kinase activity; ATP binding; metal ion binding; initiation of apoptosis; positive regulation of axon regeneration	−4.3	<0.001
*ATPase, class VI, type 11B*	*ATP11B*	ATPase activity; transmembrane movement of ions; aminophospholipid transport; cation transport;	−4.2	<0.001
*Piccolo (presynaptic cytomatrix protein)*	*PCLO*	Part of the presynaptic cytoskeletal matrix; synaptic vesicle trafficking; calcium-dependent phospholipid binding; cAMP-mediated signaling; synaptic vesicle exocytosis; insulin secretion	−4.1	0.01
*Canopy 3 homolog*	*CNPY3*	Regulates cell surface expression of immature form of TLR4; receptor binding; innate immune response	−4.0	0.01
*Tetratricopeptide repeat domain 22*	*TTC22*	Encodes protein with seven tetratricopeptide (TPR) repeats; may mediate protein-protein interactions, chaperone activity	−4.0	<0.001
*URB1 ribosome biogenesis 1 homolog*	*URB1*	Function currently unknown	−3.9	0.02
*Dynein, axonemal, heavy chain 3*	*DNAH3*	ATPase activity; nucleotide binding; ciliary or flagellar motility; microtubule-based movement	−3.8	<0.001
*Pregnancy specific beta-1-glycoprotein 3*	*PSG3*	Subgroup of the carcinoembryonic antigen (CEA) gene family; involved in adhesion recognition for several integrins; defense response	−3.8	<0.001
*Zinc finger protein 850*	*ZNF850*	DNA binding; metal ion binding; DNA-dependent transcription	−3.5	<0.002
*Lysophosphatidylcholine acyltransferase 3*	*LPCAT3*	Transferase activity, transfer of acyl groups; glycerophospholipid biosynthetic process; lipid metabolism; phosphatidylcholine acyl-chain remodeling;small molecule metabolism	−3.4	0.01

* Gene functions obtained from public databases (Entrez Gene and UniProt KB), descriptions modified due to space constraints.

† Synonymous with the false discovery rate.

**Figure 2:**
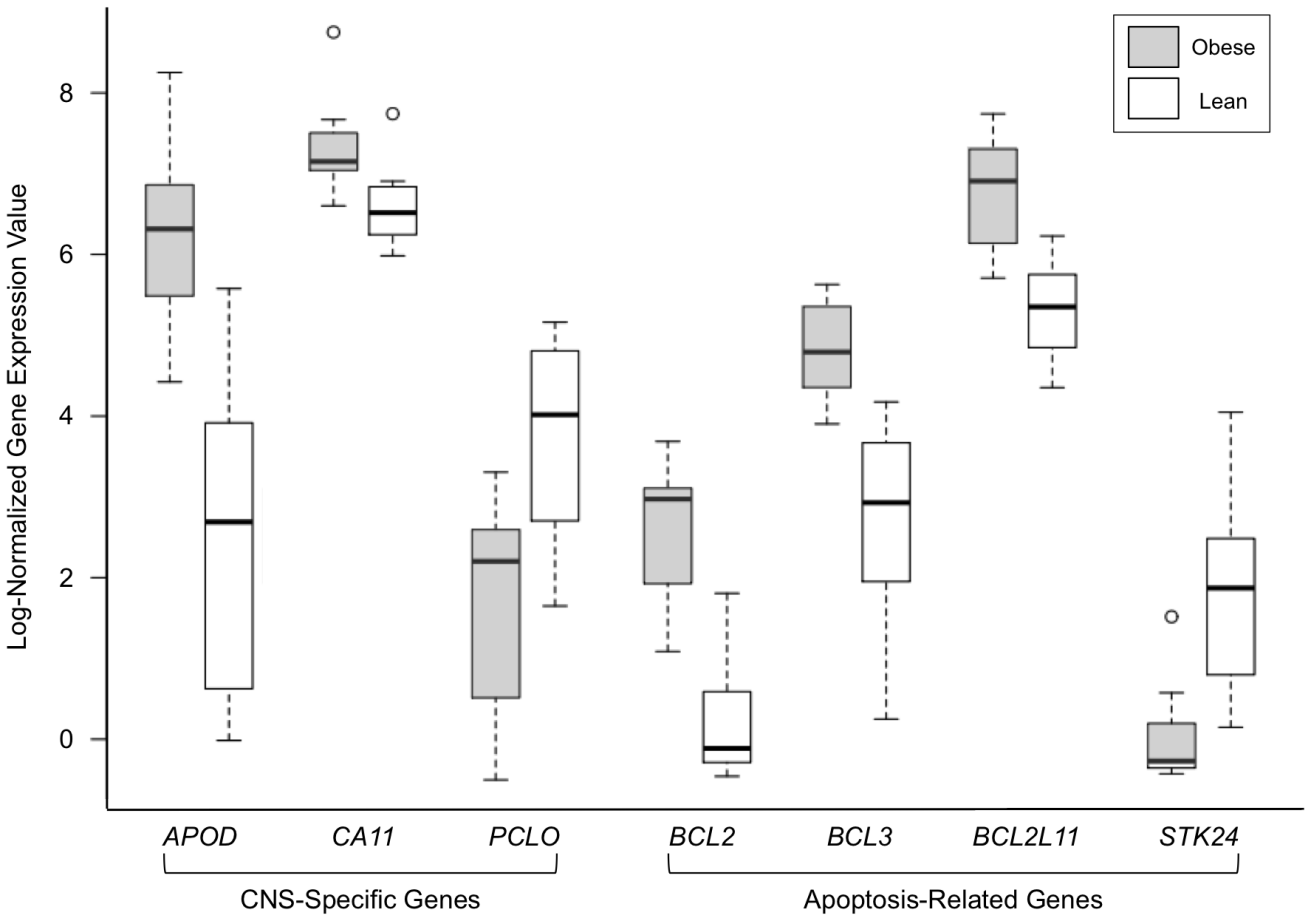
Central Nervous System-Specific and Apoptosis-Related Gene Expression Values in Obese versus Lean Subjects. Each box encompasses the interquartile range (IQR) of log-normalized gene expression values for the gene of interest in all obese (shaded box) and all lean (white box) subjects. The dark horizontal lines represent the median gene expression value for the obese or lean subjects. The whiskers represent values within 1.5 times the interquartile range greater than or less than the upper or lower quartile, respectively. The open circles represent values greater than 1.5 times the interquartile range.

Box-and-whisker plots were generated to examine the distribution of normalized gene expression data across samples (Figure S1), and to investigate the expression of genes of particular interest in obese versus lean study subjects (Figure 2). These plots demonstrate that the differences between groups are not driven by a single sample pair or by sample normalization, but indeed reflect differential expression between the obese and lean cohorts as a whole (Figure S1). Figure 2 depicts the distribution of log-normalized expression data for genes of interest, including genes tissue-specific for the central nervous system (*APOD*, *CA11*, *PCLO*), as well as those implicated in apoptotic pathways (*BCL2*, *BCL2L11*, *BCL3*, *STK24*). These boxplots provide further confirmation of the differences in gene expression in the obese and lean study populations for the CNS-specific genes, as well as for apoptosis-related genes, with clearly different median normalized gene expression values, no overlap in the interquartile ranges, and few outliers.

### Functional analysis of differentially regulated genes in fetuses of obese women

#### Molecular and cellular functions

Molecular and cellular functions that were significantly differentially regulated in fetuses of obese women are presented in Table 5A. Of these, cell death was the most significant due to multiple genes involved in apoptosis, including *B-cell CLL/lymphoma 2* (*BCL2*), *B-cell CLL/lymphoma 3* (*BCL3*), *BCL2-like 11 (BCL2L11), BCL2-like 1 (BCL2L1), serine/threonine kinase 24 (STK24)*, and *caspase 9 (CASP9)*. Anti-apoptotic genes were significantly up-regulated in fetuses of obese women (*BCL2, BCL3, BCL2L1*), while pro-apoptotic genes (*STK24, CASP9*) were down-regulated.

**Table 5:** Significantly Differentially Regulated Biological Functions and Systems in Fetuses of Obese versus Lean Women.

5A: Molecular and Cellular Functions*			5B: Physiological Systems†		
Category	P-value‡	Number of Genes	Category	P-value‡	Number of Genes
Cell Death	<0.001–0.02	25	Embryonic Development	<0.001–0.02	23
Small Molecule Biochemistry	<0.001–0.02	25	Organismal Development	<0.001–0.02	22
Cellular Function and Maintenance	<0.001–0.02	23	Tissue Development	<0.001–0.02	20
Molecular Transport	<0.001–0.02	22	Organ Development	<0.001–0.02	18
Cellular Assembly and Organization	<0.001–0.02	21	Hematological System Development and Function	<0.001–0.02	16
Cell Morphology	<0.001–0.02	15	Tumor Morphology	<0.001–0.02	8
Lipid Metabolism	<0.001–0.02	14	Endocrine System Development and Function	<0.001–0.02	4
Gene Expression	<0.001–0.02	10	Digestive System Development and Function	<0.001–0.02	4
Drug Metabolism	<0.001–0.02	7			
Cell-To-Cell Signaling and Interaction	<0.001–0.02	7			
Amino Acid Metabolism	<0.001–0.02	6			

* False discovery rate range for all categories within table 5A: 0.04–0.13.

† False discovery rate range for all categories within table 5B: 0.04–0.13.

‡ Right-tailed Fisher's exact P-value.

**Figure 3:**
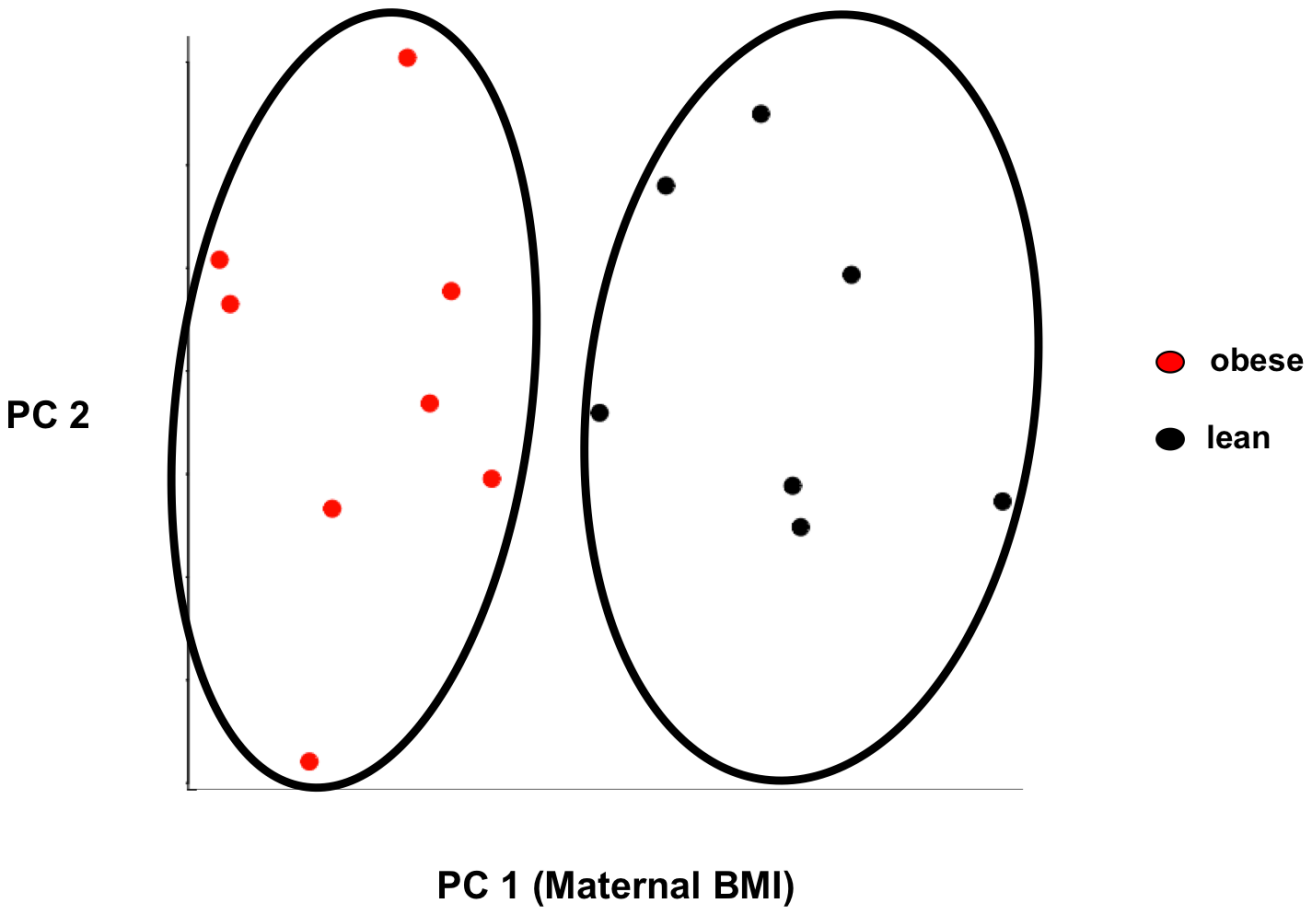
Principal Component Analysis of Genes Implicated in Central Nervous System Apoptosis. Figure demonstrates the results of the principal component analysis. Obese subjects are represented by red spheres and lean subjects are represented by black spheres. The results suggest that gene expression segregates on the basis of maternal BMI. On the x-axis is principal component (PC) 1, maternal body mass index (BMI), which accounts for the greatest proportion of variance in the gene expression data (21%). On the y-axis is PC 2, which accounts for the second greatest proportion of variance (14%).

Within the IPA category of “Cell Death,” many functional annotations associated with the central nervous system (CNS) were significantly dysregulated in fetuses of obese women. Apoptosis of cerebral cortex cells, sympathetic neurons, cortical neurons, and neuroblastoma cell lines, cell viability of dendritic cells and hippocampal neurons, and cell death of hippocampal cells were all significantly dysregulated. To investigate the association between maternal BMI and CNS apoptosis, a post hoc principal component analysis (PCA) was performed utilizing all the genes annotated by IPA to CNS apoptosis (*BCL2, BCL2L1, BCL2L11, CASP9, P4HB, REST, HMGA1, XBP1*). The PCA demonstrated that within our dataset, genes implicated in CNS apoptosis segregated primarily by maternal BMI (Figure 3).

Gene expression patterns suggestive of dysregulated apoptosis in fetuses of obese women were not limited to the nervous system. The IPA analysis predicted a significant decrease in gastrointestinal tract apoptosis, and significant dysregulation of liver cell and pancreatic beta islet cell apoptosis (Table S2). Cell death of B and T lymphocytes, embryonic stem cells, and hematopoietic progenitor cells was also significantly dysregulated (Table S3).

#### Physiological systems

Significantly differentially regulated physiological systems in fetuses of obese women are presented in Table 5B. Within these categories, functional annotations related to apoptosis, gonadogenesis and gametogenesis, and survival/cell viability of B and T lymphocytes were significantly affected. Detailed results are provided in Table S3.

#### Upstream regulators

In fetuses of obese women, the IPA upstream regulator analysis predicted the activation of estrogen receptor *(ESR1/2)* (activation Z-score 2.01), *FBJ murine osteosarcoma viral oncogene homolog (FOS)* (activation Z-score 2.17), and *signal transducer and activator of transcription 3* or *STAT3*, also called acute-phase response factor (activation Z-score 2.16). These transcriptional regulators are of particular interest because of their involvement in hormonal and inflammatory signaling pathways, leptin regulation, glucose homeostasis, and hepatic steatosis (Table 6).

**Table 6:** Activated Upstream Regulators in Fetuses of Obese versus Lean Women.

Name	Gene Function*	Predicted Activation State	Activation Z-score	Number of target genes
Estrogen Receptor (*ESR1/2*)	Estrogen signaling	Activated	2.01	5
FBJ murine osteosarcoma viral oncogene homolog (*FOS*)	Cytokine/toll-like receptor signaling; cell response to hormone signaling; leptin regulation; cell proliferation and differentiation; apoptosis	Activated	2.17	8
Signal transducer and activator of transcription 3 (*STAT3*)	Acute phase response/cytokine signaling; growth factor signaling; glucose homeostasis; cell growth and apoptosis	Activated	2.16	6

*Gene functions obtained from public databases (Entrez Gene and UniProt KB), descriptions modified due to space constraints.

### Tissue origin of differentially regulated genes

Of the 205 differentially regulated genes in fetuses of obese women, 12 were tissue-specific. Three of these 12 genes were highly expressed in CNS tissues, including *APOD*, *CA11*, and *PCLO*. Of the remaining nine genes, three mapped to the placenta, three mapped to lymphocyte populations (T- and B-cells, NK cells) or myeloid, monocyte, and dendritic cells, and one mapped to the liver. A list of the tissue-specific genes and their corresponding functions are presented in Table 7.

**Table 7:** Tissue-specific gene expression in fetuses of obese women.

Gene Name	Symbol	Tissue-Specific Expression*	Function†
*Apolipoprotein D*	*APOD*	Central Nervous System (olfactory bulb)	Encodes a component of high density lipoprotein (HDL); lipocalin involved in lipid metabolism, response to reactive oxygen species, response to axon injury
*Carbonic anhydrase XI*	*CA11*	Central Nervous System (prefrontal cortex, amygdala, cerebellum, temporal lobes, cingulate cortex)	Member of family of zinc metalloenzymes; catalyzes reversible hydration of carbon dioxide
*Piccolo (presynaptic cytomatrix protein)*	*PCLO*	Central Nervous System (prefrontal cortex, amygdala)	Part of presynaptic cytoskeletal matrix; synaptic vesicle trafficking; calcium-dependent phospholipid binding; cAMP-mediated signaling; synaptic vesicle exocytosis; insulin secretion
*Dihydroxyacetone kinase 2 homolog*	*DAK*	Liver	Member of dihydroxyacetone kinase family; glycerol metabolism; innate immune response
*Fascin homolog 3, actin-bundling protein, testicular*	*FSCN3*	Germ cell/Testis	Actin binding; spermatid development
*Sushi, von Willebrand factor type A, EGF and pentraxin domain containing 1*	*SVEP1*	Placenta	Calcium ion binding; chromatin binding and cell adhesion
*Pappalysin 2*	*PAPPA2*	Placenta	Local regulator of insulin-like growth factor availability; cell differentiation; proteolysis; regulation of cell growth
*Pregnancy specific beta-1-glycoprotein 3*	*PSG3*	Placenta	Synthesized by trophoblasts; defense response; subgroup of the carcinoembryonic antigen gene family
*NAD kinase*	*NADK*	Myeloid cells, monocytes, dendritic cells	ATP binding and kinase activity
*SP100 nuclear antigen*	*SP100*	B and T lymphocytes and NK Cells	Binds heterochromatin proteins; tumorigenesis, immunity, and gene regulation; DNA damage response; cytokine signaling
*Apolipoprotein B mRNA editing enzyme, catalytic polypeptide-like 3B*	*APOBEC3B*	B lymphocytes	DNA cytosine deamination; cellular response to virus; negative regulation of retroviral genome replication
*Killer cell lectin-like receptor subfamily C, member 3*	*KLRC3*	CD56 and NK cells	Natural killer cell signaling; carbohydrate binding to cell; cellular defense

*All genes listed are associated with tissue-specific expression >30 MoMs in BioGPS.

† Gene functions obtained from public databases (Entrez Gene and UniProt KB), descriptions modified due to space constraints.

## Discussion

In this study we demonstrated that significant differences in fetal gene expression in obese pregnant women are detectable as early as the second trimester. The two major and unexpected findings in fetuses of obese pregnant women, overexpression of *APOD* and gene expression patterns consistent with decreased brain apoptosis in the mid-trimester, suggest two potential mechanisms by which maternal obesity may lead to adverse neurodevelopmental outcomes in offspring. While prior work has demonstrated that mid-trimester amniotic fluid is enriched for brain-specific transcripts [14], [17], neither of these studies has described the types of neurological gene abnormalities demonstrated here in fetuses of obese pregnant women.

*APOD* is highly and specifically expressed in the central nervous system (CNS), and is synthesized and secreted by oligodendrocytes and astrocytes [43]. The tissue-specific mapping of *APOD* to the CNS is strongly conserved across multiple species, including human, rat and mouse [44], [45]. ApoD, the protein product, has been demonstrated to exert neuroprotective and neurotrophic effects in cell culture and in a rodent model of excitotoxic brain injury [46], [47]. However, ApoD is thought to play a role in the pathophysiology of schizophrenia [48]. Increased expression of *APOD* in the human prefrontal cortex during critical developmental periods is associated with increased susceptibility to schizophrenia [49]. Elevated levels of ApoD have also been noted in human plasma during a first psychotic episode [50], as well as in patients with bipolar disorder, Parkinson's and Alzheimer's diseases [51], [52], [53]. Cortical ApoD expression has been demonstrated to increase six- to eight-fold between the neonatal period and adulthood, with increased expression correlating with genetic and biochemical markers of oxidative stress [54]. In a rodent model, maternal high fat diet was associated with increased oxidative stress and inflammatory signaling in the brains of offspring [55], Thus, the presence of excess ApoD during brain development could itself be deleterious, or could represent a response to a harmful or neurodegenerative process.

Our results also suggest decreased apoptosis of cerebral cortex cells, and dysregulation of cell death and cell survival in multiple areas of the fetal brain. The differential gene expression observed in fetuses of obese women, with significant upregulation of *BCL2* and *BCL2L11* and downregulation of *CASP9* suggests decreased signaling through the internal or mitochondrial apoptotic pathways [56]. Animal models have similarly demonstrated differential brain gene expression and differences in brain apoptosis in offspring of obese females [57], [58]. Apoptosis plays a critical role in normal neurodevelopment [59], [60], so decreased brain apoptosis may be a potential mechanism by which maternal obesity adversely influences offspring neurodevelopment.

The IPA upstream regulator analysis predicted significant activation of the estrogen receptor (*ESR1/2*), *FOS*, and *STAT3*. Prior experience with diethylstilbestrol, a synthetic estrogen, and recent data regarding *in utero* exposure to estrogenic compounds such as bisphenol A, suggest that developmental exposure to excess estrogen signaling may result in obesity, earlier sexual maturation in girls, and increased risk for breast and other reproductive tract cancers [61], [62]. *FOS*, also called *cFOS*, is a proto-oncogene involved in inflammatory signaling, appetite regulation, and regulation of cell proliferation and apoptotic cell death [63], [64]. *FOS* has been implicated in the pathogenesis of atherosclerosis and heart disease [65], [66]. *STAT3*, also called acute-phase response factor, has been implicated in inflammatory signaling, inhibition of apoptosis, glucose homeostasis, hyperleptinemia, and hepatic steatosis [67], [68], [69], [70].

The gene *APOD* was nine-fold up-regulated in fetuses of obese women. *APOD* encodes the protein product apolipoprotein D, a member of the lipocalin family of transporter proteins and a component of high-density lipoprotein [71]. This gene is of specific interest, given its known involvement in lipid homeostasis [71], insulin resistance [72], obesity [73], and hypothalamic regulation of food intake [74]. There is mounting concern that maternal obesity, which has been associated with both intrauterine growth restriction and large-for-gestational age fetuses, may contribute to the epidemic of childhood obesity and metabolic disorders via fetal programming [75], [76]. Up-regulation of *APOD*, in conjunction with gene expression patterns consistent with increased estrogen and inflammatory signaling, suggest potential molecular mechanisms underlying the increased risk for obesity and metabolic syndrome in offspring of obese women.

The use of human amniotic fluid, rather than an animal model, to examine the effects of maternal obesity on fetal gene expression was a significant strength of this study. Other strengths include that all samples were processed by the same clinical cytogenetics and research laboratories, and that the length of sample storage was standardized, limiting any variability in specimen handling or processing techniques. Case and control samples were matched for variables known to influence gene expression, including fetal sex and gestational age. Rigorous statistical criteria were applied to identify significantly differentially regulated genes in fetuses of obese women.

One limitation of this study was its relatively small sample size. However, given the well-characterized neurodevelopmental and metabolic morbidities of offspring of obese women, and the knowledge gap regarding underlying *in utero* molecular mechanisms, we believe these pilot data are novel, and will help to focus future studies. In addition, eight matched pairs have been shown to provide near-maximal statistical stability for microarray experiments, and are well above the five subjects per group considered to be an acceptable minimum for microarray experiments [26], [77], [78]. Although maternal age was not significantly different between groups (p = 0.6), there was a three-year difference in mean age between groups (37.1±5 for obese compared to 33.9±5.5 for lean women) that may have achieved significance with larger study numbers. While this is a potential confounder, we are not aware of data suggesting that maternal age alone influences fetal gene expression in the absence of aneuploidy. The fact that all samples came from women having an amniocentesis may itself suggest underlying pregnancy co-morbidities that could theoretically introduce bias. However, amniocentesis is the only way to acquire this biofluid in living fetuses, and we excluded all fetuses with structural anomalies and/or abnormal karyotype, in order to minimize such bias. Several of the amniocenteses were performed for sonographic “soft markers” of aneuploidy, such as choroid plexus cyst and echogenic intracardiac focus. Such soft markers are not structural abnormalities and do not have clinical significance if the fetus has a euploid karyotype. These samples represent a narrow window of gestational age; this is necessarily a reflection of the time during pregnancy in which amniocentesis is routinely performed. Finally, because these amniotic fluid specimens were anonymized after collection per IRB specification, we were only able to analyze the sonographic and standard demographic data available at the time of amniocentesis, the indication for amniocentesis, and the cytogenetic result. Pregnancy outcomes and maternal medical comorbidities of included subjects were therefore unknown. However, the majority of pregnancy-related conditions that could potentially influence fetal gene expression, such as gestational diabetes and preeclampsia, would not yet exist or be diagnosed at the time of second trimester amniocentesis.

In summary, analysis of the amniotic fluid transcriptome in fetuses of obese women demonstrates gene expression patterns suggestive of decreased brain apoptosis; lipid, insulin and appetite dysregulation; and increased estrogen and inflammatory signaling. The molecular mechanisms predisposing offspring of obese women to neurodevelopmental abnormalities and metabolic complications may be initiated as early as the second trimester. While prior work has demonstrated that mid-trimester amniotic fluid is enriched for brain-specific transcripts [17], the ones demonstrated here are novel and may contribute to adverse neurodevelopmental outcomes in fetuses of obese pregnant women. Future experiments are planned to study fetal brain apoptosis, *APOD* gene expression, and offspring neurocognitive development in a mouse model of maternal diet-induced obesity.

## Supporting Information

Figure S1 **Normalized Gene Expression Values for Each Subject.** Each box encompasses the interquartile range (IQR) of log-normalized gene expression values for each microarray. Shaded boxes represent obese subjects, white boxes represent lean subjects. The dark horizontal lines represent the median gene expression value for each array. The whiskers represent values within 1.5 times the interquartile range greater than or less than the upper or lower quartile, respectively. The open circles represent values greater than 1.5 times the interquartile range.
(TIFF)

Table S1 Significantly differentially regulated genes in fetuses of obese versus lean women in the second trimester.
(DOCX)

Table S2 Significantly differentially regulated molecular and cellular functions in fetuses of obese versus lean women, with associated functional annotations.
(DOCX)

Table S3 Significantly differentially regulated physiological systems in fetuses of obese versus lean women, with associated functional annotations.
(DOCX)
