# Supplementary figures and images for: Maternal Obesity Affects Fetal Neurodevelopmental and Metabolic Gene Expression: A Pilot Study
Source: PLoS One. 2014 Feb 18;9(2):e88661. doi: 10.1371/journal.pone.0088661 (PMC3928248; doi:10.1371/journal.pone.0088661)

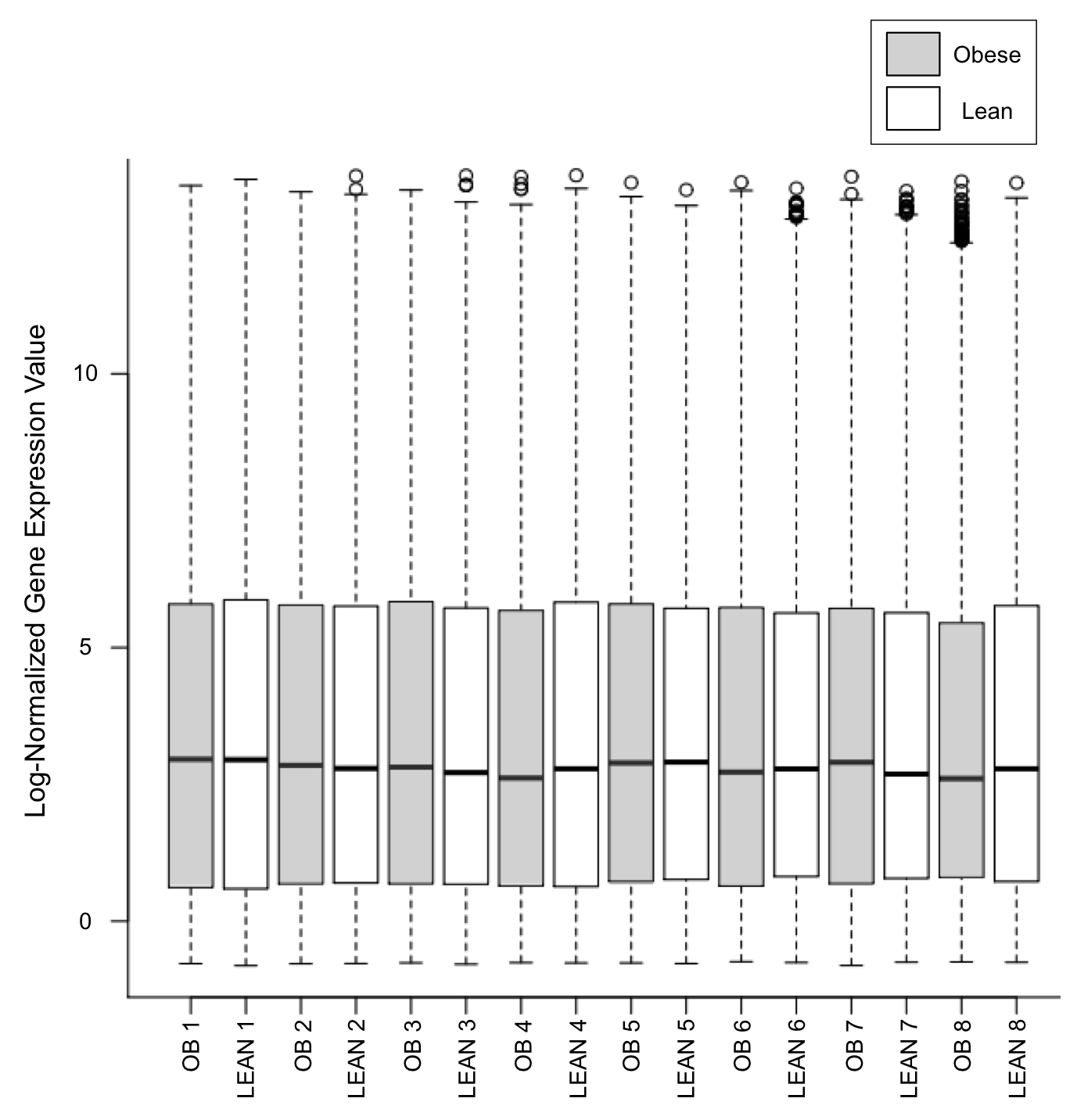

Supplement: Figure S1 — Normalized Gene Expression Values for Each Subject. Each box encompasses the interquartile range (IQR) of log-normalized gene expression values for each microarray. Shaded boxes represent obese subjects, white boxes represent lean subjects. The dark horizontal lines represent the median gene expression value for each array. The whiskers represent values within 1.5 times the interquartile range greater than or less than the upper or lower quartile, respectively. The open circles represent values greater than 1.5 times the interquartile range. (TIFF) [file pone.0088661.s001.tif]
