# Supplementary material for: Maternal Obesity Affects Fetal Neurodevelopmental and Metabolic Gene Expression: A Pilot Study
Source: PLoS One. 2014 Feb 18;9(2):e88661. doi: 10.1371/journal.pone.0088661 (PMC3928248; doi:10.1371/journal.pone.0088661)
Supplement: Table S1 — Significantly differentially regulated genes in fetuses of obese versus lean women in the second trimester. (DOCX) [file pone.0088661.s002.docx]

**Table S1: Significantly differentially regulated genes in fetuses of obese versus lean women in the second trimester**

| **Gene Name** | **Symbol** | **Affymetrix Probe ID** | **Fold Change^*^** | **BH p- value^†^** |
| --- | --- | --- | --- | --- |
| *apolipoprotein D* | *APOD* | 201525_at | 9.18 | 0.03 |
| *F-box and leucine-rich repeat protein 6* | *FBXL6* | 219189_at | 8.33 | <0.001 |
| *synaptotagmin XIII* | *SYT13* | 221859_at | 8.21 | <0.001 |
| *B-cell CLL/lymphoma 2* | *BCL2* | 203685_at | 7.62 | <0.001 |
| *matrix metallopeptidase 9 (gelatinase B, 92kDa gelatinase, 92kDa type IV collagenase)* | *MMP9* | 203936_s_at | 5.80 | <0.001 |
| *chromosome X open reading frame 56* | *CXorf56* | 235442_at | 5.06 | 0.01 |
| *MORN repeat containing 1* | *MORN1* | 222933_at | 5.01 | 0.01 |
| *zinc finger protein 551* | *ZNF551* | 211721_s_at | 4.65 | 0.01 |
| *zinc finger protein 483* | *ZNF483* | 243770_at | 4.32 | <0.001 |
| *B-cell CLL/lymphoma 3* | *BCL3* | 204907_s_at | 4.27 | <0.001 |
| *glycolipid transfer protein domain containing 2* | *GLTPD2* | 230971_x_at | 4.21 | 0.03 |
| *uncharacterized LOC348120* | *LOC348120* | 231132_at | 3.99 | <0.001 |
| *insulin-like growth factor binding protein-like 1* | *IGFBPL1* | 227760_at | 3.79 | <0.001 |
| *uncharacterized LOC283352* | *LOC283352* | 1561757_a_at | 3.65 | 0.03 |
| *long intergenic non-protein coding RNA 161* | *LINC00161* | 1552466_x_at | 3.62 | 0.03 |
| *ADAMTS-like 1* | *ADAMTSL1* | 229585_at | 3.43 | 0.02 |
| *transcription factor AP-2 epsilon (activating enhancer binding protein 2 epsilon)* | *TFAP2E* | 1555348_at | 3.39 | <0.001 |
| *Sp2 transcription factor* | *SP2* | 211736_at | 3.38 | <0.001 |
| *rhophilin, Rho GTPase binding protein 1* | *RHPN1* | 235998_at | 3.35 | <0.001 |
| *ATP-binding cassette, sub-family D (ALD), member 1* | *ABCD1* | 205142_x_at | 3.12 | <0.001 |
| *pleckstrin homology domain containing, family G (with RhoGef domain) member 5* | *PLEKHG5* | 227142_at | 3.08 | <0.001 |
| *zinc finger protein 426* | *ZNF426* | 205964_at | 3.08 | 0.01 |
| *syntrophin beta 2 (dystrophin- associated protein A1, 59kDa, basic component 2)* | *SNTB2* | 213814_s_at | 2.93 | <0.001 |
| *pleckstrin homology-like domain, family B, member 3* | *PHLDB3* | 1557948_at | 2.88 | 0.04 |
| *autophagy related 9B* | *ATG9B* | 229252_at | 2.85 | <0.001 |
| *zinc finger protein 800* | *ZNF800* | 227097_at | 2.74 | <0.001 |
| *tripeptidyl peptidase I* | *TPP1* | 214196_s_at | 2.67 | <0.001 |
| *flavin containing monooxygenase 4* | *FMO4* | 206263_at | 2.64 | 0.01 |
| ***Gene Name*** | **Symbol** | **Affymetrix Probe ID** | **Fold Change^*^** | **BH p- value^†^** |
| *ER membrane protein complex subunit 10* | *EMC10* | 224727_at | 2.62 | 0.02 |
| *islet cell autoantigen 1, 69kDa* | *ICA1* | 207949_s_at | 2.62 | <0.001 |
| *BCL2-like 11 (apoptosis facilitator)* | *BCL2L11* | 1553096_s_at | 2.61 | 0.01 |
| *RAS-like, family 11, member A* | *RASL11A* | 238353_at | 2.60 | <0.001 |
| *v-Ki-ras2 Kirsten rat sarcoma viral oncogene homolog* | *KRAS* | 1559204_x_at | 2.59 | <0.001 |
| *fascin homolog 3, actin-bundling protein, testicular* | *FSCN3* | 220379_at | 2.57 | 0.02 |
| *extracellular leucine-rich repeat and fibronectin type III domain containing 2* | *ELFN2* | 1563108_at | 2.48 | 0.05 |
| *doublesex and mab-3 related transcription factor 2* | *DMRT2* | 223704_s_at | 2.45 | 0.03 |
| *TAF10 RNA polymerase II, TATA box binding protein (TBP)-associated factor, 30kDa* | *TAF10* | 235906_at | 2.40 | <0.001 |
| *X-box binding protein 1* | *XBP1* | 242021_at | 2.33 | <0.001 |
| *SPRY4 intronic transcript 1 (non-protein coding)* | *SPRY4-IT1* | 1566968_at | 2.27 | 0.01 |
| *lens intrinsic membrane protein 2, 19kDa* | *LIM2* | 220519_s_at | 2.23 | 0.01 |
| *synaptotagmin-like 3* | *SYTL3* | 238423_at | 2.22 | <0.001 |
| *spastic paraplegia 11 (autosomal recessive)* | *SPG11* | 1560214_at | 2.22 | <0.001 |
| *transmembrane protein 40* | *TMEM40* | 219503_s_at | 2.22 | <0.001 |
| *NAD kinase* | *NADK* | 208918_s_at | 2.20 | <0.001 |
| *adaptor-related protein complex 4, sigma 1 subunit* | *AP4S1* | 235647_at | 2.19 | 0.02 |
| *sushi, von Willebrand factor type A, EGF and pentraxin domain containing 1* | *SVEP1* | 213247_at | 2.18 | 0.02 |
| *chloride channel, voltage-sensitive 4* | *CLCN4* | 217556_at | 2.18 | <0.001 |
| *transmembrane protein 138* | *TMEM138* | 223113_at | 2.16 | 0.02 |
| *Boc homolog* | *BOC* | 224184_s_at | 2.15 | <0.001 |
| *DTX2P1-UPK3BP1-PMS2P11 readthrough (non-protein coding)* | *DTX2P1-UPK3BP1-PMS2P11* | 179_at | 2.12 | <0.001 |
| *dihydroxyacetone kinase 2 homolog* | *DAK* | 218688_at | 2.12 | <0.001 |
| *derlin 3* | *DERL3* | 228897_at | 2.10 | <0.001 |
| *protein tyrosine phosphatase type IVA, member 3* | *PTP4A3* | 206574_s_at | 2.09 | 0.01 |
| *transient receptor potential cation channel, subfamily V, member 6* | *TRPV6* | 1559405_a_at | 2.09 | <0.001 |
| **Gene Name** | **Symbol** | **Affymetrix Probe ID** | **Fold Change^*^** | **BH p- value^†^** |
| *signal sequence receptor, delta pseudogene 1* | *SSR4P1* | 223768_at | 2.08 | 0.03 |
| *iduronate 2-sulfatase* | *IDS* | 217432_s_at | 2.08 | <0.001 |
| *guanine nucleotide binding protein (G protein), alpha 11 (Gq class)* | *GNA11* | 221955_at | 2.08 | 0.04 |
| *bridging integrator 3* | *BIN3* | 1557582_at | 2.06 | 0.02 |
| *family with sequence similarity 129, member B* | *FAM129B* | 233974_s_at | 2.05 | <0.001 |
| *presenilin associated, rhomboid-like* | *PARL* | 228881_at | 2.05 | <0.001 |
| *EPS8-like 2* | *EPS8L2* | 229020_x_at | 2.02 | <0.001 |
| *G protein-coupled receptor 3* | *GPR3* | 214613_at | 2.02 | <0.001 |
| *zinc finger protein 562* | *ZNF562* | 219163_at | 2.00 | <0.001 |
| *zinc finger and BTB domain containing 10* | *ZBTB10* | 235726_at | 1.98 | <0.001 |
| *oleoyl-ACP hydrolase* | *OLAH* | 222945_x_at | 1.97 | 0.05 |
| *BCL2-like 1* | *BCL2L1* | 206665_s_at | 1.95 | 0.02 |
| *poliovirus receptor-related 4* | *PVRL4* | 223540_at | 1.95 | <0.001 |
| *Na+/K+ transporting ATPase interacting 4* | *NKAIN4* | 230668_at | 1.95 | 0.05 |
| *MYC-associated zinc finger protein (purine-binding transcription factor)* | *MAZ* | 229807_s_at | 1.90 | 0.02 |
| *tetratricopeptide repeat domain 3* | *TTC3* | 1569472_s_at | 1.88 | 0.03 |
| *Uncharacterized LOC100506489* | *LOC100506489* | 1559670_at | 1.86 | 0.01 |
| *Phosphogluconate dehydrogenase* | *PGD* | 201118_at | 1.85 | 0.01 |
| *SCY1-like 2* | *SCYL2* | 221220_s_at | 1.84 | 0.04 |
| *F-box protein 34* | *FBXO34* | 218539_at | 1.82 | 0.02 |
| *SON DNA binding protein* | *SON* | 201086_x_at | 1.81 | <0.001 |
| *zinc finger and BTB domain containing 7B* | *ZBTB7B* | 205853_at | 1.81 | 0.04 |
| *sestrin 2* | *SESN2* | 223195_s_at | 1.80 | 0.04 |
| *zinc finger,AN1-type domain 3* | *ZFAND3* | 222493_s_at | 1.79 | <0.001 |
| *nuclear mitotic apparatus protein 1* | *NUMA1* | 214251_s_at | 1.76 | 0.01 |
| *solute carrier family 35, member E2B* | *SLC35E2B* | 230694_at | 1.76 | <0.001 |
| *NEDD4 binding protein 1* | *N4BP1* | 48612_at | 1.76 | <0.001 |
| *Uncharacterized LOC100506235* | *LOC100506235* | 232364_at | 1.76 | <0.001 |
| *EH-domain containing 1* | *EHD1* | 209038_s_at | 1.75 | 0.04 |
| *carbonic anhydrase XI* | *CA11* | 209726_at | 1.75 | <0.001 |
| *N(alpha)-acetyltransferase 15, NatA auxiliary subunit* | *NAA15* | 222836_at | 1.75 | 0.01 |
| *kelch-like 9* | *KLHL9* | 213117_at | 1.73 | <0.001 |
| **Gene Name** | **Symbol** | **Affymetrix Probe ID** | **Fold Change^*^** | **BH p- value^†^** |
| *transducin-like enhancer of split 4* | *TLE4* | 235765_at | 1.66 | <0.001 |
| *haloacid dehalogenase-like hydrolase domain containing3* | *HDHD3* | 221256_s_at | 1.60 | 0.03 |
| *threonyl-tRNA synthetase* | *TARS* | 240206_at | 1.59 | 0.02 |
| *uncharacterized LOC100506083* | *LOC100506083* | 1568787_at | 1.57 | 0.03 |
| *cyclin-dependent kinase 13* | *CDK13* | 214287_s_at | 1.55 | 0.01 |
| *protein tyrosine phosphatase, non-receptor type 23* | *PTPN23* | 223149_s_at | 1.55 | <0.001 |
| *high mobility group AT-hook 1* | *HMGA1* | 210457_x_at | 1.55 | <0.001 |
| *fucosyltransferase 6 (alpha (1,3) fucosyltransferase)* | *FUT6* | 210399_x_at | 1.53 | 0.03 |
| *Desumoylating isopeptidase 1* | *DESI1* | 212527_at | 1.52 | <0.001 |
| *ADP-ribosylation factor 6* | *ARF6* | 203311_s_at | 1.50 | 0.01 |
| *zer-1 homolog* | *ZER1* | 202452_at | 1.50 | 0.05 |
| *DCP1 decapping enzyme homolog A* | *DCP1A* | 218508_at | 1.50 | 0.04 |
| *polymerase (DNA-directed), delta interacting protein 3* | *POLDIP3* | 215357_s_at | 1.48 | 0.02 |
| *protein phosphatase 1, regulatory (inhibitor) subunit 11* | *PPP1R11* | 1566303_s_at | 1.47 | 0.03 |
| *mitochondrial ribosomal protein L37* | *MRPL37* | 222993_at | 1.47 | <0.001 |
| *HYDIN2, axonemal central pair apparatus protein (pseudogene)* | *HYDIN2* | 1554907_a_at | 1.45 | 0.02 |
| *zinc finger and BTB domain containing 7A* | *ZBTB7A* | 226554_at | 1.43 | <0.001 |
| *ubiquitin-conjugating enzyme E2Q family member 1* | *UBE2Q1* | 217978_s_at | 1.43 | 0.03 |
| *prolyl 4-hydroxylase, beta polypeptide* | *P4HB* | 1564494_s_at | 1.42 | <0.001 |
| *DnaJ (Hsp40) homolog, subfamily B, member 11* | *DNAJB11* | 223054_at | 1.41 | <0.001 |
| *ataxin 2-like* | *ATXN2L* | 228767_at | 1.41 | <0.001 |
| *nuclear receptor coactivator 1* | *NCOA1* | 209105_at | 1.39 | 0.01 |
| *regulator of G-protein signaling 14* | *RGS14* | 211021_s_at | 1.38 | 0.02 |
| *ARP2 actin-related protein 2 homolog* | *ACTR2* | 200728_at | 1.37 | <0.001 |
| *ADP-ribosylation factor 1* | *ARF1* | 208750_s_at | 1.28 | 0.03 |
| *amino-terminal enhancer of split* | *AES* | 217729_s_at | 1.27 | 0.01 |
| *actin related protein 2/3 complex, subunit 5, 16kDa* | *ARPC5* | 1555797_a_at | 1.22 | <0.001 |
| *Morf4 family associated protein 1* | *MRFAP1* | 226091_s_at | -1.17 | 0.02 |
| **Gene Name** | **Symbol** | **Affymetrix Probe ID** | **Fold Change^*^** | **BH p- value^†^** |
| *DEAD (Asp-Glu-Ala-Asp) box polypeptide 3, X-linked* | *DDX3X* | 201210_at | -1.23 | <0.001 |
| *zinc finger protein 641* | *ZNF641* | 226509_at | -1.25 | 0.04 |
| *COP9 constitutive photomorphogenic homolog subunit 6* | *COPS6* | 201405_s_at | -1.31 | <0.001 |
| *protein tyrosine phosphatase, non-receptor type 18 (brain-derived)* | *PTPN18* | 213521_at | -1.34 | <0.001 |
| *RAN binding protein 1* | *RANBP1* | 221915_s_at | -1.37 | <0.001 |
| *dead end homolog 1* | *DND1* | 222070_at | -1.37 | <0.001 |
| *Snf2-related CREBBP activator protein* | *SRCAP* | 213667_at | -1.40 | <0.001 |
| *SP100 nuclear antigen* | *SP100* | 202864_s_at | -1.40 | 0.01 |
| *CSE1 chromosome segregation 1-like* | *CSE1L* | 201111_at | -1.41 | 0.02 |
| *methylcrotonoyl-CoA carboxylase 2 (beta)* | *MCCC2* | 209624_s_at | -1.42 | 0.02 |
| *RE1-silencing transcription factor* | *REST* | 204535_s_at | -1.44 | <0.001 |
| *lysine (K)-specific demethylase 3B* | *KDM3B* | 210878_s_at | -1.45 | <0.001 |
| *primase, DNA, polypeptide 2 (58kDa)* | *PRIM2* | 205628_at | -1.46 | <0.001 |
| *PAP associated domain containing 5* | *PAPD5* | 229043_at | -1.51 | <0.001 |
| *dopamine receptor D3* | *DRD3* | 211625_s_at | -1.51 | 0.05 |
| *GCN1 general control of amino-acid synthesis 1-like 1* | *GCN1L1* | 212139_at | -1.51 | 0.01 |
| *proteasome (prosome, macropain) subunit, alpha type, 4* | *PSMA4* | 203396_at | -1.51 | 0.02 |
| *chromosome 17 open reading frame 79* | *C17orf79* | 225096_at | -1.52 | 0.04 |
| *general transcription factor IIIA* | *GTF3A* | 201338_x_at | -1.53 | 0.04 |
| *guanine nucleotide binding protein (G protein), q polypeptide* | *GNAQ* | 224861_at | -1.56 | 0.01 |
| *long intergenic non-protein coding RNA 623* | *LINC00623* | 222001_x_at | -1.57 | 0.05 |
| *heterogeneous nuclear ribonucleoprotein A3* | *HNRNPA3* | 211932_at | -1.57 | 0.01 |
| *developmental pluripotency associated 4* | *DPPA4* | 241199_x_at | -1.57 | <0.001 |
| *uncharacterized LOC730098* | *LOC730098* | 230327_at | -1.61 | 0.01 |
| *nuclear casein kinase and cyclin-dependent kinase substrate 1* | *NUCKS1* | 222027_at | -1.63 | 0.01 |
| **Gene Name** | **Symbol** | **Affymetrix Probe ID** | **Fold Change^*^** | **BH p- value^†^** |
| *zinc finger protein 410* | *ZNF410* | 209944_at | -1.63 | 0.01 |
| *apolipoprotein B mRNA editing enzyme, catalytic polypeptide-like 3B* | *APOBEC3B* | 206632_s_at | -1.64 | 0.02 |
| *MARVEL domain containing 1* | *MARVELD1* | 223095_at | -1.66 | 0.01 |
| *SYF2 homolog, RNA splicing factor* | *SYF2* | 202553_s_at | -1.70 | 0.01 |
| *RNA binding motif protein 20* | *RBM20* | 238763_at | -1.72 | <0.001 |
| *centrobin, centrosomal BRCA2 interacting protein* | *CNTROB* | 228633_s_at | -1.74 | 0.03 |
| *pappalysin 2* | *PAPPA2* | 213332_at | -1.76 | <0.001 |
| *PNN-interacting serine/arginine-rich protein* | *PNISR* | 212179_at | -1.79 | <0.001 |
| *relaxin 2* | *RLN2* | 214519_s_at | -1.82 | 0.04 |
| *nei endonuclease VIII-like 2* | *NEIL2* | 226585_at | -1.82 | <0.001 |
| *v-ets erythroblastosis virus E26 oncogene homolog 1* | *ETS1* | 224833_at | -1.82 | 0.05 |
| *mago-nashi homolog 2 (pseudogene)* | *MAGOH2* | 217693_x_at | -1.86 | 0.04 |
| *chondroadherin-like* | *CHADL* | 1557123_a_at | -1.89 | 0.03 |
| *myotubularin related protein 6* | *MTMR6* | 214429_at | -1.90 | 0.01 |
| *RAB12, member RAS oncogene family* | *RAB12* | 239891_x_at | -1.91 | 0.01 |
| *regenerating islet-derived 1 alpha* | *REG1A* | 209752_at | -1.92 | <0.001 |
| *killer cell lectin-like receptor subfamily C, member 3* | *KLRC3* | 207723_s_at | -1.95 | <0.001 |
| *mediator complex subunit 30* | *MED30* | 227786_at | -1.96 | <0.001 |
| *parvin, alpha* | *PARVA* | 213675_at | -2.02 | 0.02 |
| *potassium channel tetramerisation domain containing 15* | *KCTD15* | 222664_at | -2.03 | <0.001 |
| *Moesin* | *MSN* | 200600_at | -2.03 | 0.03 |
| *choroideremia-like (Rab escort protein 2)* | *CHML* | 226350_at | -2.05 | <0.001 |
| *polo-like kinase 1 substrate 1* | *PLK1S1* | 233241_at | -2.08 | 0.02 |
| *zinc finger CCCH-type, antiviral 1-like* | *ZC3HAV1L* | 228280_at | -2.09 | <0.001 |
| *polymerase (DNA directed), gamma* | *POLG* | 203366_at | -2.10 | 0.04 |
| *MEF2 activating motif and SAP domain containing transcriptional regulator* | *MAMSTR* | 1557091_at | -2.12 | 0.01 |
| *pleckstrin homology domain containing, family A member 6* | *PLEKHA6* | 229245_at | -2.12 | <0.001 |
| *chromosome 10 open reading frame 40* | *C10orf40* | 1556648_a_at | -2.15 | <0.001 |
| *uncharacterized LOC100130776* | *LOC100130776* | 1555907_at | -2.15 | 0.01 |
| **Gene Name** | **Symbol** | **Affymetrix Probe ID** | **Fold Change^*^** | **BH p- value^†^** |
| *RAD9-HUS1-RAD1 interacting nuclear orphan 1* | *RHNO1* | 225837_at | -2.17 | <0.001 |
| *glutathione synthetase* | *GSS* | 211630_s_at | -2.18 | 0.02 |
| *zinc finger protein 780B* | *ZNF780B* | 214899_at | -2.29 | 0.03 |
| *F-box and leucine-rich repeat protein 18* | *FBXL18* | 227500_at | -2.30 | <0.001 |
| *excision repair cross-complementing rodent repair deficiency, complementation group 1 (includes overlapping antisense sequence)* | *ERCC1* | 228131_at | -2.31 | 0.04 |
| *SWI/SNF related, matrix associated, actin dependent regulator of chromatin, subfamily a, member 5* | *SMARCA5* | 213859_x_at | -2.34 | <0.001 |
| *chromosome 1 open reading frame 212* | *C1orf212* | 238640_at | -2.34 | 0.02 |
| *SEC23 interacting protein* | *SEC23IP* | 209176_at | -2.41 | <0.001 |
| *uncharacterized LOC283516* | *LOC283516* | 1557345_at | -2.41 | 0.03 |
| *leucine rich repeat containing 38* | *LRRC38* | 1558375_at | -2.44 | 0.03 |
| *chromosome 6 open reading frame 58* | *C6orf58* | 238385_at | -2.46 | <0.001 |
| *protein tyrosine phosphatase-like (proline instead of catalytic arginine), member A* | *PTPLA* | 219654_at | -2.46 | <0.001 |
| *caspase 9, apoptosis-related cysteine peptidase* | *CASP9* | 240437_at | -2.51 | <0.001 |
| *COBW domain containing 1* | *CBWD1* | 229804_x_at | -2.53 | <0.001 |
| *zinc finger protein 880* | *ZNF880* | 235913_at | -2.58 | 0.02 |
| *structural maintenance of chromosomes 2* | *SMC2* | 213253_at | -2.62 | <0.001 |
| *methylenetetrahydrofolate reductase (NAD(P)H)* | *MTHFR* | 217071_s_at | -2.71 | 0.01 |
| *spire homolog 2* | *SPIRE2* | 1568916_at | -2.71 | 0.03 |
| *SR-related CTD-associated factor 11* | *SCAF11* | 235579_at | -2.74 | 0.03 |
| *ADAM metallopeptidase with thrombospondin type 1 motif, 15* | *ADAMTS15* | 1553427_at | -2.78 | 0.03 |
| *membrane-spanning 4-domains, subfamily A, member 1* | *MS4A1* | 228592_at | -2.91 | <0.001 |
| *carboxylesterase 5A* | *CES5A* | 1553465_a_at | -2.92 | 0.01 |
| *amyloid beta precursor protein (cytoplasmic tail) binding protein 2* | *APPBP2* | 202630_at | -2.93 | 0.01 |
| *mediator complex subunit 4* | *MED4* | 217843_s_at | -3.12 | 0.02 |
| *3-ketodihydrosphingosine reductase* | *KDSR* | 229850_at | -3.31 | 0.04 |
| **Gene Name** | **Symbol** | **Affymetrix Probe ID** | **Fold Change^*^** | **BH p- value^†^** |
| *lysophosphatidylcholine acyltransferase 3* | *LPCAT3* | 213615_at | -3.38 | 0.01 |
| *zinc finger protein 850* | *ZNF850* | 244640_at | -3.46 | <0.001 |
| *pregnancy specific beta-1-glycoprotein 3* | *PSG3* | 211741_x_at | -3.80 | <0.001 |
| *dynein, axonemal, heavy chain 3* | *DNAH3* | 1560803_at | -3.81 | <0.001 |
| *URB1 ribosome biogenesis 1 homolog* | *URB1* | 212996_s_at | -3.91 | 0.02 |
| *tetratricopeptide repeat domain 22* | *TTC22* | 220309_at | -3.95 | <0.001 |
| *canopy 3 homolog* | *CNPY3* | 228369_at | -3.97 | 0.01 |
| *piccolo (presynaptic cytomatrix protein)* | *PCLO* | 213558_at | -4.06 | 0.01 |
| *ATPase, class VI, type 11B* | *ATP11B* | 1554556_a_at | -4.24 | <0.001 |
| *serine/threonine kinase 24* | *STK24* | 215188_at | -4.33 | <0.001 |
| *uncharacterized LOC100286925* | *LOC100286925* | 240115_at | -4.66 | <0.001 |

^*^ Positive fold change means the gene is up-regulated in fetuses of obese compared to lean women, negative fold change means the gene is down-regulated in fetuses of obese women

^†^ Synonymous with the false discovery rate
