# Supplementary material for: Maternal Obesity Affects Fetal Neurodevelopmental and Metabolic Gene Expression: A Pilot Study
Source: PLoS One. 2014 Feb 18;9(2):e88661. doi: 10.1371/journal.pone.0088661 (PMC3928248; doi:10.1371/journal.pone.0088661)
Supplement: Table S2 — Significantly differentially regulated molecular and cellular functions in fetuses of obese versus lean women, with associated functional annotations. (DOCX) [file pone.0088661.s003.docx]

**Table S2: Significantly differentially regulated molecular and cellular functions in fetuses of obese versus lean women, with associated functional annotations**

| **Category** | **Functional Annotation** | **P-value^*^** | **False Discovery Rate^†^** | **Bias-corrected z-score** | **Number of Molecules** |
| --- | --- | --- | --- | --- | --- |
| Cell Death | apoptosis of sympathetic neuron | <0.001 | 0.04 | -1.11 | 4 |
| Cell Death | cell viability of dendritic cells | <0.001 | 0.04 | 0.92 | 4 |
| Cell Death | cell viability of hippocampal neurons | 0.002 | 0.08 |  | 3 |
| Cell Death | apoptosis of cerebral cortex cells | 0.002 | 0.09 | -2.17 | 5 |
| Cell Death | apoptosis of neuroblastoma cell lines | 0.004 | 0.10^‡^ | -1.94 | 5 |
| Cell Death | apoptosis of cortical neurons | 0.006 | 0.10 | -1.96 | 4 |
| Cell Death | cell death of striatal neurons | 0.009 | 0.10 |  | 3 |
| Cell Death | cell death of hippocampal cells | 0.01 | 0.10 | -1.92 | 4 |
| Cell Death | apoptosis of colon cancer cell lines | <0.001 | 0.04 | -2.72 | 9 |
| Cell Death | apoptosis of gastrointestinal cells | 0.006 | 0.10 |  | 3 |
| Cell Death | apoptosis of liver | <0.001 | 0.04 | -1.00 | 9 |
| Cell Death | apoptosis of liver cells | <0.001 | 0.04 | -0.88 | 8 |
| Cell Death | apoptosis of hepatocytes | 0.002 | 0.08 | -1.16 | 6 |
| **Category** | **Functional Annotation** | **P-value^*^** | **False Discovery Rate^†^** | **Bias-corrected z-score** | **Number of Molecules** |
| Cell Death | apoptosis of B cell lymphoma cells | <0.001 | 0.04 |  | 3 |
| Cell Death | apoptosis of lymphoma cell lines | 0.002 | 0.08 | -0.68 | 7 |
| Cell Death | cell viability of B-lymphocyte derived cell lines | 0.002 | 0.08 |  | 4 |
| Cell Death | apoptosis of B lymphocytes | 0.006 | 0.10 | -0.24 | 5 |
| Cell Death | apoptosis of leukocyte cell lines | 0.008 | 0.10 | -1.27 | 6 |
| Cell Death | apoptosis of B-lymphocyte derived cell lines | 0.007 | 0.10 | -1.01 | 5 |
| Cell Death | cell viability of T lymphocytes | 0.005 | 0.10 | 1.85 | 5 |
| Cell Death | anoikis of epithelial cells | <0.001 | 0.04 |  | 3 |
| Cell Death | anoikis | 0.003 | 0.10 | -1.22 | 5 |
| Cell Death | cell death of cancer cells | 0.007 | 0.10 | -0.16 | 8 |
| Cell Death | killing of tumor cell lines | 0.001 | 0.08 |  | 4 |
| Cell Death | apoptosis of embryonic stem cells | <0.001 | 0.04 | 0.11 | 4 |
| Cell Death | apoptosis of melanoma cell lines | <0.001 | 0.04 | -0.69 | 6 |
| Cell Death | apoptosis of myeloma cell lines | <0.001 | 0.04 | -1.18 | 5 |
| Cell Death | delay in apoptosis | 0.001 | 0.07 | -0.01 | 4 |
| Cell Death | apoptosis of mammary tumor cells | <0.001 | 0.08 |  | 3 |
| **Category** | **Functional Annotation** | **P-value^*^** | **False Discovery Rate^†^** | **Bias-corrected z-score** | **Number of Molecules** |
| Cell Death | apoptosis of muscle cell lines | <0.001 | 0.08 | 0.30 | 4 |
| Cell Death | apoptosis of beta islet cells | 0.002 | 0.09 |  | 4 |
| Cell Death | apoptosis of embryonic cell lines | 0.002 | 0.09 | -1.21 | 7 |
| Cell Death | cell death of embryonic cells | 0.005 | 0.10 | 0.43 | 5 |
| Cell Death | apoptosis of tumor cells | 0.007 | 0.10 | -1.20 | 8 |
| Cell Death | apoptosis of cancer cells | 0.004 | 0.10 | -0.55 | 7 |
| Cell Death | cell death of lung cancer cell lines | 0.004 | 0.10 | -0.68 | 7 |
| Cell Death | apoptosis of chondrocytes | 0.004 | 0.10 |  | 3 |
| Cell Death | cell viability of thymocytes | 0.004 | 0.10 |  | 3 |
| Cell Death | apoptosis of gonadal cells | 0.005 | 0.10 |  | 6 |
| Cell Death | apoptosis of splenocytes | 0.006 | 0.10 |  | 3 |
| Cell Death | apoptosis of germ cells | 0.007 | 0.10 |  | 5 |
| Cell Death | cell viability of epithelial cells | 0.008 | 0.10 |  | 3 |
| Gene Expression | initiation of transcription of RNA | <0.001 | 0.04 |  | 6 |
| Cell Morphology | permeabilization of mitochondria | 0.001 | 0.07 | -1.22 | 4 |
| Cellular Function and Maintenance | organization of nucleus | 0.002 | 0.09 |  | 5 |
| Cellular Function and Maintenance | organization of mitotic spindle | 0.004 | 0.10 |  | 3 |
| **Category** | **Functional Annotation** | **P-value^*^** | **False Discovery Rate^†^** | **Bias-corrected z-score** | **Number of Molecules** |
| Lipid Metabolism | quantity of phosphatidylinositol 4,5-diphosphate | 0.001 | 0.07 |  | 3 |
| Molecular Transport | transport of protein | 0.005 | 0.10 |  | 9 |
| Small Molecule Biochemistry | hydrolysis of nucleotide | 0.001 | 0.07 | -1.19 | 6 |
| Small Molecule Biochemistry | hydrolysis of GTP | 0.004 | 0.10 |  | 4 |
| Cell-To-Cell Signaling and Interaction | sensitization of tumor cell lines | <0.001 | 0.04 |  | 3 |
| Cell-To-Cell Signaling and Interaction | sensitization of cells | <0.001 | 0.04 |  | 4 |
| Cellular Assembly and Organization | stabilization of mitochondria | <0.001 | 0.04 |  | 3 |

^*^ Right-tailed Fisher’s exact p-value

^†^ Synonymous with BH-p value

^‡^ All false-discovery rate values of 0.10 are equal to 0.0965, but have been rounded to 0.10
