# Supplementary material for: Maternal Obesity Affects Fetal Neurodevelopmental and Metabolic Gene Expression: A Pilot Study
Source: PLoS One. 2014 Feb 18;9(2):e88661. doi: 10.1371/journal.pone.0088661 (PMC3928248; doi:10.1371/journal.pone.0088661)
Supplement: Table S3 — Significantly differentially regulated physiological systems in fetuses of obese versus lean women, with associated functional annotations. (DOCX) [file pone.0088661.s004.docx]

**Table S3:** **Significantly differentially regulated physiological systems in fetuses of obese versus lean women, with associated functional annotations**

| **Category** | **Functional Annotation** | **P-value^*^** | **False discovery rate^†^** | **Number of Genes** |
| --- | --- | --- | --- | --- |
| Tumor Morphology | cell death of lymphoma cells | <0.001 | 0.04 | 4 |
| Tumor Morphology | sensitization of tumor cells | <0.001 | 0.04 | 3 |
| Tumor Morphology | apoptosis of B cell lymphoma cells | <0.001 | 0.04 | 3 |
| Tumor Morphology | apoptosis of mammary tumor cells | 0.002 | 0.08 | 3 |
| Tumor Morphology | apoptosis of melanoma cells | 0.002 | 0.08 | 3 |
| Tumor Morphology | cell death of cancer cells | 0.007 | 0.10^‡^ | 8 |
| Tumor Morphology | apoptosis of tumor cells | 0.007 | 0.10 | 8 |
| Embryonic Development | apoptosis of embryonic stem cells | <0.001 | 0.04 | 4 |
| Embryonic Development | gametogenesis | 0.001 | 0.07 | 12 |
| Embryonic Development | apoptosis of embryonic cell lines | 0.002 | 0.09 | 7 |
| Embryonic Development | gonadogenesis | 0.003 | 0.10 | 13 |
| Embryonic Development | spermatogenesis | 0.004 | 0.10 | 10 |
| Embryonic Development | development of testis | 0.004 | 0.10 | 5 |
| Embryonic Development | oogenesis | 0.004 | 0.10 | 3 |
| Tissue Development | accumulation of B lymphocytes | 0.001 | 0.08 | 3 |
| **Category** | **Functional Annotation** | **P-value^*^** | **False discovery rate^†^** | **Number of Genes** |
| Hematological System Development and Function | survival of pro-B lymphocytes | <0.001 | 0.04 | 3 |
| Hematological System Development and Function | cell viability of B-lymphocyte derived cell lines | 0.002 | 0.08 | 4 |
| Hematological System Development and Function | cell viability of thymocytes | 0.004 | 0.10 | 3 |
| Hematological System Development and Function | quantity of double-negative T lymphocyte | 0.005 | 0.10 | 4 |
| Hematological System Development and Function | cell viability of T lymphocytes | 0.005 | 0.10 | 5 |
| Hematological System Development and Function | quantity of pre-B lymphocytes | 0.005 | 0.10 | 5 |
| Hematological System Development and Function | cell viability of hematopoietic progenitor cells | 0.006 | 0.10 | 4 |

^*^ Right-tailed Fisher’s exact p-value

^†^ Synonymous with BH-p value

^‡^ All false-discovery rate values of 0.10 are equal to 0.0965, but have been rounded to 0.10
